# The Effect of Sumac (*Rhus coriaria *L.)Powder on Serum Glycemic Status, ApoB, ApoA-I and Total Antioxidant Capacity in Type 2 Diabetic Patients 

**Published:** 2014

**Authors:** Farzad Shidfar, Seyedeh Tayebeh Rahideh, Asadollah Rajab, Nafise Khandozi, Sharieh Hosseini, Shahrzad Shidfar, Faraz Mojab

**Affiliations:** a Research Institute for Islamic and Complementary Medicine, Iran University of Medical Sciences, Tehran, Iran.; b Iranian Diabetes Association, Tehran, Iran.; c Islamic Azad University, Robat Karim Branch, Robat Karim, Iran.; d University of Massachusetts, Worcester Memorial Hospital, Worcester, Massachusetts, USA.; e School of Pharmacy, Shahid Beheshti University of Medical Sciences, Tehran, Iran.

**Keywords:** Sumac, Glycemic status, Apolipoprotein, Total antioxidant capacity, Diabetes

## Abstract

Sumac (*Rhus coriaria *L.) is used as an herbal remedy in traditional medicine. The aim of this study was to determine the effects of sumac (*R. coriaria*) on serum glycemic status, apolipoprotein (apo) B, apoA-I and total antioxidant capacity (TAC) in type 2 diabetic patients. This double blind randomized controlled clinical trial was conducted on 41type 2 diabetic volunteers randomly assigned into 3g/day sumac powder (n=22) or placebo (n=19) groups over 3 months. Blood samples were collected before and after the intervention. Serum glucose and HbA1c were measured using enzymatic and turbidimetric inhibition immunoassay methods, respectively. ApoB, apoA-I and TAC were determined using turbidimetric immunoassay and spectrophotometric methods, respectively. There were signiﬁcant decreases in serum glucose and HbA1c and also apoB levels at the end of study compared with initial values (*P*< 0.0001, *P*= 0.002 and *P*< 0.0001,respectively). Also, there was a significant difference in HbA1c and TAC levels between placebo and sumac groups at the end of study (*P*< 0.05).In sumac group, there were significant increase in apoA-I and TAC(*P*< 0.0001) compared with initial values. The mean of differences of serum glucose, HbA1c, apoB, apoA-I, apoB/apoA-I ratio and TAC between groups were significant (*P*< 0.05). In conclusion, these results showed the favorite effect of sumac consumption on serum glycemic status, apoB, apoA-I and TAC levels in in type 2 diabetic patients.

## Introduction

According to WHO reports, 180 million people suffer from diabetes worldwide (1). It is estimated that developing countries in Asia and in the Middle East, particularly in Persian Gulf states, will have the largest increase in the prevalence of diabetes by 2030 (2). The number of Iranian diabetic patients who are 25-64 years old is 7.7 percent and half of them are unaware of their condition and also 6.8 percent of adult Iranians have impaired fasting glucose (3). Abnormalities in carbohydrate, lipid and lipoprotein metabolism lead to hyperglycemia and many complications such hyperlipidemia, hyperinsulinemia, hypertension and atherosclerosis (4). Therefore glycemic and lipids profiles and also oxidative stress control is fundamental for the management of diabetes.

Spices and herbal remedies have been used since ancient times to treat a variety of disorders (5). Sumac is the common name for a genus (*Rhus*) that contains over 250 individual species of ﬂowering plants in the family Anacardiaceae (6). This genus is found in temperate and tropical regions worldwide, with representative members by geographic location. This spice comes from the berries of a wild bush that grow in all Mediterranean areas (7). Sumac is widely used in Turkey, Iran and Arab countries as a condiment, either in pure form or in combination with other spices(6, 8). In Persian traditional medicine, sumac (*Rhus coriaria *L.) is believed to have atheroprotective effects and is consumed in some Persian dishes (7). In Iran, *R.coriaria* is traditionally used as a table spice especially along with rich dishes and is highly recommended for adjustment of the blood lipids in diabetic patients(9). It is used as an herbal remedy in traditional medicine owing to its anti-ﬁbrogenic, antifungal, anti-inﬂammatory, antimalarial, antimicrobial, antimutagenic, antioxidant, antithrombin, antitumorigenic, antiviral, cytotoxic, hypoglycaemic, leukopaenic (6) and atheroprotective effects (10). Sumac contains various substances, phenol acids and flavonoids, such as gallic acid (GA), methyl gallate, kaempferol, and quercetin (11). Gallic acid possesses potent antioxidant properties (12). Also, Sumac is known to be a rich source of hydrolysable tannins (10). Tannin and its derivatives are strong antioxidants (7). *In-vitro* and *in-vivo* studies have shown that tannins have anticarcinogenic effect (7, 12).

Sumac has been found to possess hypoglycaemic and hypolipidemic properties but evidence for its effects is mainly based on the results that are obtained from *in-vitro *experiments and animal studies and we didn't find any human study about the effect of sumac on them. One of our primitive concerns is that extrapolating it to humans *in-vivo*. Mohammadi *et al.* reported a single dose of administration of the extract of *R. coriaria *fruits reduced postprandial blood glucose (PBG) significantly in rats (9). However, in other study*, *addition of sumac to the diet of rabbits had no effect on blood glucose (12). Sumac has reduced total cholesterol (TC), low density lipoprotein(LDL-C),triglyceride (TG) and blood sugar in animal studies (9,13,14). 

Prolonged hyperglycemia by means of the increased oxidative stress was suggested as the major cause of endothelial dysfunction, which plays a pivotal role in development of atherosclerosis (15). A number of *in-vitro *and animal investigations were published concerning antioxidant effects of sumac (8, 12,16). The only a human study was published by Chacraborty *et al*. Over all, the results of their study showed for the ﬁrst time that the spice sumac is a potent antioxidant which protects humans against oxidative DNA-damage and suggest that GA is may be account for sumac effects (8). 

Recent researches have focused on new factors including apoB and apoA-I that these factors have a role in the formation of atherosclerosis. ApoB and apoA-I are more important than the LDL and high density lipoprotein (HDL) particles as a marker of atherogenic risk (17, 18). We didn’t find any human study about the effect of sumac on glycemic status, apolipoproteins and total antioxidant capacity (TAC). In this present study, we have investigated the effects of sumac (*R.coriaria*) on serum glycemic status, apoB, apoA-I and TAC in type 2 diabetic patients. 

## Experimental

*Materials and methods*

This double blind randomized controlled clinical trial was conducted on 41type 2 diabetic volunteers (age: 20-60 years, HbA1c 6-8%, at least 2 years duration of diabetes) who wereall non-smokers, no alcohol and nonusers of antioxidant supplements at least 3 months before the intervention participated and were randomly allocated either to the placebo group or to the sumac group. All of them signed consent form before the start of the study. The study was approved by the Ethics Committee of the Tehran University of Medical Science. Exclusion criteria were the patients reluctance to continue working with, a change in the type or dose of medication, changes in diet or physical activity daily, taking any antioxidant supplement, consuming less than 80% of supplements delivered to the patient at the baseline, recent receiving insulin, pregnancy and lactation, kidney, liver, thyroid diseases and anemia.

During the intervention, each participant consumed 3.0 g sumac powder daily over 3 months. The participants of the placebo group received the same amount of lactose (19). The dietary intake of patients evaluated with 24-hour dietary recall questionnaire over 3 days at the beginning and end of study. These completed through telephone interviews to all participants. Also, anthropometric measurements assessed before and after study. Physical activity was measured by an International Physical Activity Questionnaire (2) at baseline and at the end of the intervention. Blood samples (7 mL) were collected in tubes immediately before and after the intervention under fasting condition (12-14 h). After serum preparation, serum glucose was measured using enzymatic method by commercial kit (Elitech, France) which performed on Hitachi 717 auto analyzer. HbA1c was determined using turbidimetric inhibition immunoassay method with commercial kit (Roche, Germany) on the Cobas. ApoB and apoAI were measured using turbidimetric immunoassay method with commercial kit (Roche, Germany) on the Cobas. TAC was determined using spectrophotometric method as described by Miller and Rice-Evans (20). 

*Statistical analysis*

All analyses were performed using SPSS statistical software (version 16, SPSS). Normality of variables was assessed by Kolmogorov–Smirnov tests. The differences were assessed by the Student’s t-test. Differences between groups were compared with independent samples t-test. Differences within groups (before and after intervention) were compared with paired samples t-test. Serum glucose was compared by the Wilcoxon’s test. We also calculated the mean of differences (before and after intervention) in serum glucose, HbA1c, apoB, apoA-I, TAC levels apoB/apoA-I ratio. The data are presented as means ± standard deviation. Differences were compared for statistical signiﬁcance at the level *P*< 0.05.

## Results

There were no statistical differences with regard to age, duration of diabetes, weight, BMI, waist circumferences (Table 1) and physical activity between sumac (*Rhus coriaria L.*) and placebo groups. Also, energy and nutrients intake were not significantly different between groups at baseline (Table 2) and did not change during the study.

**Table 1:** Baseline characteristics in sumac and placebo groups

**Variable **	**Sumac group ** **(n=22)**	**Placebo group ** **(n=19)**	**p-values **
Age(year)	46.1±8.52	47.5±8.4	0.33
Weight(Kg)	80.1±12.5	77.5±13.2	0.42
BMI(Kg/m^2^)	29.5±2.8	29.5±2.2	0.95
Waist Circumferences(cm)	97.9±8.8	97.9±9.05	0.1
Duration of Diabetes(year)	5.4±3.5	5.1±3.42	0.62

Data presented as mean ± standard deviation.

**Table 2:** Baseline energy and nutrients intake in sumac and placebo groups

**Variable **	**Sumac group ** **(n=22)**	**Placebo group ** **(n=19)**	**p-values **
Energy Intake(Kcal)	1579.6±735	1287.9±485	0.09
Total Carbohydrate(g)	231.7±149.8	174.1±75.5	0.064
Total Protein(g)	66.3±20.7	53.4±23.8	0.07
Total Fat(g)	46.6.6±31.01	35.1.6±14.4	0.07

Data presented as mean ± standard deviation.

*Effect of sumac powder on* *glycemic status:*

Results of serum glucose and HbA1c levels are presented in Table 3. Serum glucose was decreased significantly after the intervention compared with baseline (*P*< 0.0001). The mean of differences were signiﬁcant between groups (*P*< 0.0001).

Signiﬁcant decrease (*P*= 0.002) in HbA1c in sumac group compare with initial values between groups. There was signiﬁcant difference in HbA1c at the end of study between the two groups (*P*= 0.003) and also the mean of differences were signiﬁcant between groups (*P*= 0.002).

**Table3:** Serum glucose, HbA1c, apoB, apoA-I, apoB/apoA-I ratio and TAC in sumac and placebo groups at baseline and after the intervention

**Biochemical parameters**	**Sumac group** **(n=22)**			**Placebo group** **(n=19)**		
	**Before **	**After **	**p** **-** **values**	**Before **	**After **	**p** **-** **values**
glucose (mg/dl)	150.14 ± 42.03	130.55 ± 40.55	0.000	155.47 ± 81.83	157.10 ± 85.09	0.048
HbA1c (%)	6.75 ± 1.2	6.18 ± 0.83*	0.002	7.30 ± 1.31	7.31 ± 1.32*	0.609
apoB (mg/dl)	90.45 ± 16.38	80.03 ± 16.22	0.000	87.79 ± 22.61	90.02 ± 20.53	0.137
apoA-I (mg/dl)	119.36 ± 19.69	140.36 ± 28.95*	0.000	119.46 ± 3.69	118.85 ± 3.89*	0.028
apoB/apoA-I ratio	0.77 ± 0.15	0.59 ± 0.14*	0.000	0.74 ± 0.19	0.76 ± 0.17*	0.100
TAC (μmol/L)	2.05 ± 0.79	2.85 ± 0.90*	0.000	2.34 ± 0.69	2.30 ± 0.60*	0.581

The values shown are the mean ± SD (standard deviation).

* Values (after) were signiﬁcantly different between groups,* P*< 0.05 (independent samples t-test).

*Effect of sumac powder on* *apoB, apoA-I* *and* *apoB/apoA-I* *ratio*

As shown in Table 3, in the sumac group, there was a signiﬁcant decrease in apoB (*P*< 0.0001), a signiﬁcant increase in apoA-I (*P*< 0.0001) and a signiﬁcant decrease in the apoB/apoA-I ratio (*P*< 0.0001) after intervention compared with before intervention. There were significant differences in relation to apoA-I and apoB/apoA-I ratio after intervention between groups (*P*= 0.002 and *P*= 0.001, respectively) (Table 3). The mean of differences of apoB, apoA-I and apoB/apoA-I ratio between groups were significant (*P*< 0.0001).

*Effect of sumac powder on* *TAC*

As shown in Table 3, TAC was increased significantly after the intervention compared with baseline (*P*< 0.0001), no such effect were observed in the placebo group. There was signiﬁcant difference in TAC at the end of study between the two groups (*P*= 0.026) and also the mean of differences were signiﬁcant between groups (*P*< 0.0001).

## Discussion

In the present study, the mean of differences of serum glucose, HbA1c,apoB, apoA-I, apoB/apoA-I ratio and TAC between groups were significant (*P*< 0.05).

The evidence for the effect of sumac on blood glucose is mainly based on the results that are obtained from *in-vitro *and *in-vivo* in animal studies and we didn't find any human study about the effect of sumac on glycemic status. The results of the present investigation are similar to the findings of* in-vitro *and animal studies (9, 21). Mohammadi *et al*. reported a single dose of administration of the extract of *R. coriaria *fruits reduced PBG significantly in rats and in the long term experiment, on the day of 21, PBG was found to be significantly lower (by 26%)that is compared to diabetic control group.Oral Glucose Tolerance Test (OGTT) results and the ability of inhibiting the α-glucosidases activities by the extract indicate that control of postprandial glucose level might be mediated through the inhibition of carbohydrate digestion or absorption. Also, antihyperglycemic effects of *R.coriaria* fruits may be related to modulation of insulin (INS) secretion or action because no change in the INS and glucose transporter type-4 (GLUT-4) genes expression was described (9). It has already been reported that *in*-*vitro* hypoglycemic activity of the ethyl acetate extract of fruits of *R. coriaria* is due to inhibition of α-amylase(21). Sumac contains various substances, phenol acids and flavonoids, such as gallic acid, methyl gallate, kaempferol, and quercetin (11), that one or all of them may be responsible for the hypoglycaemic activity of sumac (22). The presence of phenolic acids, such as gallic acid, methyl gallate, or protocatechuic acid in *R. coriaria* support the folkloric use of this plant as spice, food preserving as well as wound cleaning(11). It has been established that quercetin promotes normalization of the level of glycemia and reduces high blood serum concentrations of cholesterol and low density lipoproteins, seen in diabetes (23). This effect probably occurred with inhibition of intestinal absorption of glucose through the GLUT-2(24). However, in one study*, R. coriaria *had no effect on glucose in diabetic animals (12). *R. verniciflua* extract suppressed effectively the increase of blood glucose level during treatment in diabetic rats (13). Perhaps in these two previous studies, dose of sumac or duration of study wasn't enough to cause significant changes in the blood glucose.

Type 2 diabetes mellitus is associated with a high risk for coronary heart disease (CHD). A variety of lipoprotein and apolipoprotein ratios have been proposed that may reflect the balance of cholesterol delivery and removal at the arterial wall and provide an assessment of CHD risk (25). Apolipoproteins are important structural and functional proteins in lipoprotein particles, which transport lipids. Recent reports from prospective risk studies indicate that the apoB/apoA-I ratio, which reﬂects the cholesterol balance between potentially atherogenic and antiatherogenic lipoprotein particles, is a useful predictor of risk of both non-fatal and fatal myocardial infarction and it has been reported to be a better predictor of CV risk than any of the cholesterol indices (26, 27). In the US population, apolipoprotein measurements signiﬁcantly predict CHD death, independently of conventional lipids and other CV risk factors (smoking, dyslipidaemia, hypertension, obesity, diabetes and C-reactive protein) (27). Furthermore, the predictive ability of apo Bitself for detecting CHD death was better than any of the routine clinical lipid measurements (27). In the present study, the mean of differences of apoB, apoA-I and apoB/apo A-I ratio between groups were significant (*P*< 0.05). The total value of apoB indicates the number of potentially atherogenic lipoproteins. ApoA-I is important in removing excess cholesterol from tissues and incorporating it into HDL for reverse transport to the liver. The ratio of apoB/apoA-I hence reﬂects the balance of cholesterol transport, so the higher the value, the higher the propensity for cholesterol deposition, and consequently the higher the risk for atherogenesis (26). In our study, signiﬁcant decrease in the apoB/apoA-I ratio and the mean of differences before and after intervention were seen in sumac group significantly (*P*< 0.05), but there weren't significant in placebo group. Evidence from various studies indicates that many herbal medicinal products have potential hypocholesterolaemic activity. The results were obtained from Ho *et al*. studies claimed that glycoprotein isolated from *R. verniciﬂua* fruit for two weeks resulted a signiﬁcant decrease in plasma total cholesterol, LDL-C and TG (14) that these results were similar to the results of Mohammadi *et al*. study (9). This present study is probably the ﬁrst study documenting a positive effect of sumac on apolipoproteins level in human. Therefore, more experiments should be performed on human trials to prove this idea.

In the present study, the antioxidant ability of sumac was proved by measuring TAC that increased significantly after the intervention compared with baseline (*P*< 0.0001). Differences and the mean of differences between placebo group and sumac groups were significant (*P*< 0.05). Different extracts of *R. verniciﬂua* exhibited strong antioxidant activity. Many studies have suggested that extract of *Rhus coriaria L.* fruits may be a source of natural antioxidants (7). Evidence for this effect is based on results obtained in *in-vitro* experiment (16) and animal study (9, 12, 13, 14). The results of Chacraborty *et al*. study showed for the ﬁrst time that the spice sumac is a potent antioxidant which protects humans against oxidative DNA-damage and suggest that GA is may be account for sumac effects (8).Sumac contains various substances and exactly tannin and gallic acids may be responsible for the antioxidant ability of this plant. Tannic acid extracted from *Rhus chinensis Mill. *could effectively scavenge the O^-2^. Glycoprotein isolated from *R. verniciﬂua* stokes fruit improved the antioxidant levels (12). *R. verniciflua* stokes (RVS)contains six major low molecular compounds as *p*-coumaric acid, fustin, kaempferol-3-*O*-glucoside, sulfuretin, butein and kaempferol that this extract could contribute to the antioxidant activities and inhibition of intracellular ROS level (28). Further studies are required to show the most active constituent of sumac fruit.

In conclusion, sumac consumption may have favorite effect on glycemic status, apoB, apoA-I, TAC levels and apoB/apoA-I ratio and may decrease CVD risk in type 2 diabetic patients. Further studies are required to show the active constituent(s) of sumac*.*

This present study is probably the ﬁrst study documenting the effect of sumac on apolipoproteins levels in human.
